# Flies from L.A., The Sequel: A further twelve new species of *Megaselia* (Diptera: Phoridae) from the BioSCAN Project in Los Angeles (California, USA)

**DOI:** 10.3897/BDJ.4.e7756

**Published:** 2016-04-14

**Authors:** Emily A. Hartop, Brian V. Brown, R. Henry L. Disney

**Affiliations:** ‡ Natural History Museum of Los Angeles County, Los Angeles, United States of America; § University of Cambridge, Cambridge, United Kingdom

**Keywords:** Diptera, Phoridae, urban biodiversity

## Abstract

**Background:**

Presented are continued results from the BioSCAN Project, an urban biodiversity study sampling primarily from private backyards in Los Angeles, California (USA).

Presented are continued results from the BioSCAN Project, an urban biodiversity study sampling primarily from private backyards in Los Angeles, California (USA).

**New information:**

Twelve new species of *Megaselia* (Diptera: Phoridae) are described: *M. baileyae*, *M. friedrichae*, *M. gonzalezorum*, *M. joanneae*, *M. losangelensis*, *M. phyllissunae*, *M. pongsaiae*, *M. shatesae*, *M. stoakesi*, *M. studentorum*, *M. voluntariorum*, *M. wongae*.

## Introduction

In spite of extensive taxonomic work, urban biodiversity is still largely unknown in most metropolitan areas. This makes big finds inevitable, and big finds are big news; the description of 30 new species and one new Nearctic record from the BioSCAN project in urban Los Angeles (Hartop et al. 2015) prompted extensive media coverage. Major news outlets worldwide, from NBC to The New Yorker, picked up on the news. On social media, the story “went viral”. Suddenly, three dipterists working on an obscure group of flies were catapulted into the closest thing they may ever know to fame. It was clear from all the "buzz" (pun intended) that there is a lot of public interest in the biodiversity of the urban environments. The authors hope that this interest, and the corresponding media hulaballoo, helps get the message out: there is an enormous taxonomic deficiency, including (or, perhaps, especially) in rapidly changing urban environments, and we need people, funding, and institutions to get behind identifying, describing, and monitoring new species now. Baseline collections of urban fauna must be established in the present if there is hope for understanding the introductions and extinctions that will occur in the future. The BioSCAN Project, an ongoing urban biodiversity study of insects in Southern California, is doing just that (Brown et al. 2014, Hartop 2014).

Truthfully, the authors were not surprised to find dozens of new species in Los Angeles: this was a predicted problem and opportunity (Hartop et al. (2015). The original paper did not tell the whole story, however, as it was based on only 10,000 phorids collected over the first 3 months of the BioSCAN project. After an entire year of sampling, the project yielded a total of 43,651 phorids and 68 species of *Megaselia*. 43 of those species, 68%, were (or are herein) described as new to science from the BioSCAN project.

Describing the final twelve species from year one of BioSCAN required the authors to dig deeper into the collections of old, poorly preserved type material: a persistent thorn in the side of the *Megaselia* taxonomist. Sorting out all of the errors and misidentifications is a slow, frustrating process (Disney 1983, Hartop et al. 2016). Dedication to photographing, dissecting, and slide mounting historic types is the only way to make progress on this genus. Often paratype series are found to be useless for anything beyond demonstrating the limitations of historical definitions of species complexes. The series are often composed of any number of radically different species, with some being the opposite sex of the holotype and matched to the species based only on superficial characters. Despite the frustrations of study of this genus, the remarkable diversity of biologies of these flies makes them a varied and essential group to document in any ecosystem (Disney 1994).

## Materials and methods

Specimens were collected by Townes lightweight style Malaise traps (Townes 1972) purchased from Sante Traps, Lexington, Kentucky. Traps were set up at thirty sites in Los Angeles (CA: USA) (Hartop et al. 2015: table 1). Specimens were captured and preserved in 95% ethanol. Methods for dissection and specific mounting protocol followed those recommended for this genus (Disney 2009). Specimens selected as holotypes, and some of the paratypes, were dissected and slide mounted by first clearing in clove oil and then mounting in Canada Balsam. Some paratypes and additional material were mounted directly in Berlese’s fluid purchased from D. J. & D. Henshaw, Waltham Abbey, England and then sealed with dammar varnish (a commercially available product used for preserving artwork).

Unlike the first set of new species described from the project, hypandria were not dissected out and photographed. The species herein described are all rare flies in the survey and are represented sometimes only by a single specimen, and never more than ten (out of over 43,000 specimens). This led the authors to decide against sacrificing specimens to potentially damaging genital dissection. Also due to the rarity of specimens, habitus photos of dried specimens are not available for all species, as some of the flies were slide mounted for identification before they were determined to be new.

Specimens were examined using a Leica M205C stereo microscope and an Olympus BX40 compound microscope. Photography was done with a Keyence VHX-5000 digital microscope. Specimens are deposited in the Natural History Museum of Los Angeles County, USA (LACM) and the Cambridge University Museum of Zoology, UK (CUMZ).

Following the authors’ previously established system, descriptions are presented as tables supplemented by habitus and wing photographs, genitalia drawings, and additional images of any salient features (Hartop and Brown 2014). Clarification of some of the characters for this system can be found in Hartop et al. (2015), along with the first thirty new species described from the BioSCAN project. With this manuscript, we present a single change to our system: we are removing the “relative posterior setation” character from our description tables. Relative posterior setation is often so close in length that a curved seta can cause discrepancies between two viewers of the same genitalia, so we think it better to let the genitalia illustrations (that clearly show all setae, including those on T6) inform the reader.

## Data resources

An annotated list of literature necessary for the identification of Nearctic *Megaselia* is given in Hartop et al. (2015). A single addition to the Nearctic fauna is found in Hartop et al. (2016). Primary keys used for Nearctic *Megaselia* are those of Borgmeier (Borgmeier 1964, Borgmeier 1966) in his revisions of the North American fauna, although all world literature was considered when identifying these species.

A web-based key for *Megaselia* is in progress by authors Hartop and Brown. With a group as large and taxonomically difficult as this genus, dichotomous keys are often cumbersome, frustrating, and largely useless to anyone not well versed in the group. The online key that is being created will include a system to rapidly narrow down potential matches based on key characters, and then allow the user to further narrow species choices visually based on high quality habitus and wing photos and clear illustrations of the male genitalia. This key should be available online by the end of 2016.

## Taxon treatments

### Megaselia baileyae

Hartop, Brown, & Disney 2016 sp. n.

urn:lsid:zoobank.org:act:E9DCFE7D-59EF-430E-B574-152014D3BB8B

#### Materials

- **Type status:** Holotype. **Occurrence:** catalogNumber: 322013; recordedBy: Hoffman; individualCount: 1; sex: male; lifeStage: adult; **Taxon:** kingdom: Animalia; phylum: Arthropoda; class: Insecta; order: Diptera; family: Phoridae; genus: Megaselia; specificEpithet: baileyae; scientificNameAuthorship: Hartop, Brown, & Disney; **Location:** country: USA; stateProvince: California; municipality: Los Angeles; locality: Glendale; **Event:** samplingProtocol: Malaise trap; verbatimEventDate: 28.VI-5.VII.2014; **Record Level:** institutionCode: LACM; collectionCode: ENT
- **Type status:** Paratype. **Occurrence:** catalogNumber: 322014; recordedBy: Hoffman; individualCount: 1; sex: male; lifeStage: adult; **Taxon:** kingdom: Animalia; phylum: Arthropoda; class: Insecta; order: Diptera; family: Phoridae; genus: Megaselia; specificEpithet: baileyae; scientificNameAuthorship: Hartop, Brown, & Disney; **Location:** country: USA; stateProvince: California; municipality: Los Angeles; locality: Glendale; **Event:** samplingProtocol: Malaise trap; verbatimEventDate: 3-9.V.2014; **Record Level:** institutionCode: LACM; collectionCode: ENT
- **Type status:** Paratype. **Occurrence:** catalogNumber: 322015; recordedBy: Hoffman; individualCount: 1; sex: male; lifeStage: adult; **Taxon:** kingdom: Animalia; phylum: Arthropoda; class: Insecta; order: Diptera; family: Phoridae; genus: Megaselia; specificEpithet: baileyae; scientificNameAuthorship: Hartop, Brown, & Disney; **Location:** country: USA; stateProvince: California; municipality: Los Angeles; locality: Glendale; **Event:** samplingProtocol: Malaise trap; verbatimEventDate: 26.IX-4.X.2014; **Record Level:** institutionCode: LACM; collectionCode: ENT
- **Type status:** Paratype. **Occurrence:** recordedBy: Hoffman; individualCount: 1; sex: male; lifeStage: adult; **Taxon:** kingdom: Animalia; phylum: Arthropoda; class: Insecta; order: Diptera; family: Phoridae; genus: Megaselia; specificEpithet: baileyae; scientificNameAuthorship: Hartop, Brown, & Disney; **Location:** country: USA; stateProvince: California; municipality: Los Angeles; locality: Glendale; **Event:** samplingProtocol: Malaise trap; verbatimEventDate: 28.VI-5.VII.2014; **Record Level:** institutionCode: CUMZ
- **Type status:** Paratype. **Occurrence:** recordedBy: Hoffman; individualCount: 1; sex: male; lifeStage: adult; **Taxon:** kingdom: Animalia; phylum: Arthropoda; class: Insecta; order: Diptera; family: Phoridae; genus: Megaselia; specificEpithet: baileyae; scientificNameAuthorship: Hartop, Brown, & Disney; **Location:** country: USA; stateProvince: California; municipality: Los Angeles; locality: Glendale; **Event:** samplingProtocol: Malaise trap; verbatimEventDate: 26.IX-4.X.2014; **Record Level:** institutionCode: CUMZ
- **Type status:** Other material. **Occurrence:** recordedBy: Hoffman; individualCount: 2; sex: male; lifeStage: adult; **Taxon:** kingdom: Animalia; phylum: Arthropoda; class: Insecta; order: Diptera; family: Phoridae; genus: Megaselia; specificEpithet: baileyae; scientificNameAuthorship: Hartop, Brown, & Disney; **Location:** country: USA; stateProvince: California; municipality: Los Angeles; locality: Glendale; **Event:** samplingProtocol: Malaise trap; verbatimEventDate: V-VI.2014; **Record Level:** institutionCode: LACM; collectionCode: ENT

#### Description

**Figure 1.**
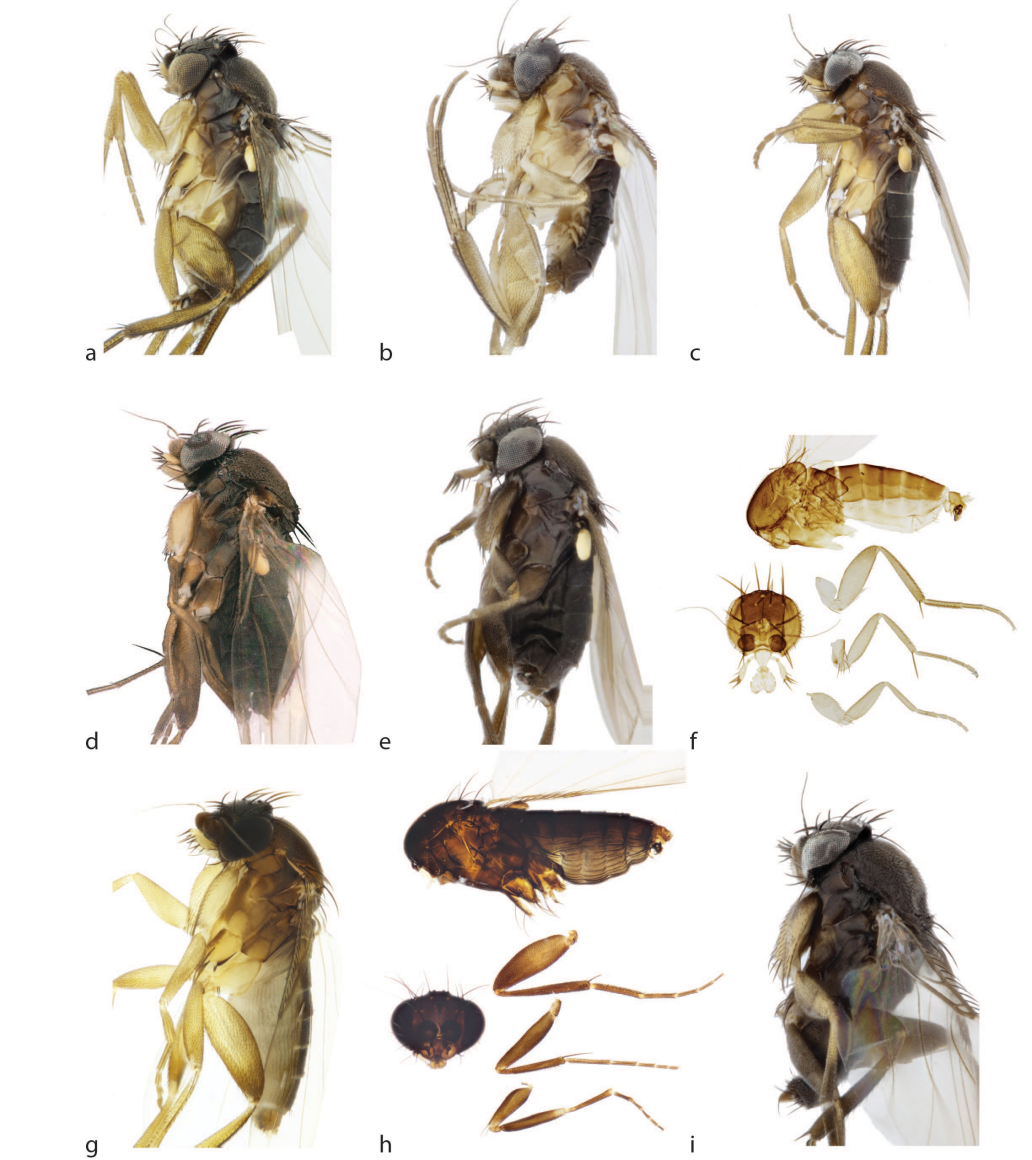
Habitus images (left lateral). a. *Megaselia baileyae*. b. *Megaselia friedrichae*. c. *Megaselia gonzalezorum*. d. *Megaselia joanneae*. e. *Megaselia losangelensis*. f. *Megaselia phyllissunae*. g. *Megaselia pongsaiae*. h. *Megaselia shatesae*. i. *Megaselia stoakesi*.

See description Table 1 and Fig. 1a, Fig. 3a, Fig. 4a.

#### Diagnosis

Male. In the group VIII key of Borgmeier (1966), *M. baileyae* keys to *M. pygmaeoides* [now considered to be *M. berndseni* (Schmitz 1919)], to which it is very similar. The most notable difference between these similar species is in the overall lighter coloration of *M. baileyae*, which has legs that are clearly yellow, not light brown like *M. berndseni*. This subtle difference in coloration is easily confirmed by looking at the hind femur, which is largely yellow but has a dark brown spot apically. This contrasts with the femur of *M. berndseni* which, although it does darken slightly apically, is clearly a light, mottled brown throughout. Details of the genitalia also differ between the species although there, too, they are quite similar. In contrast to the brown, rounded left process of the hypandrium found in *M. berndseni*, *M. baileyae* has a left lobe that is large, pale and ends abruptly, appearing almost as if it has been broken or cut (Fig. 4a). This lobe is so light, in fact, that you can see parts of the penis-complex through the lobe (visible as darkened areas in Fig. 4a).

#### Etymology

Named in honor of Kelsey Bailey, BioSCAN Photographer, for her hard work on all our publications and outreach.

#### Distribution

Los Angeles, California (USA).

#### Biology

Unknown.

### Megaselia friedrichae

Hartop, Brown, & Disney 2016 sp. n.

urn:lsid:zoobank.org:act:E901838F-5A40-44A5-B005-D368B94DCF4B

#### Materials

- **Type status:** Holotype. **Occurrence:** catalogNumber: 322024; recordedBy: Hogg; individualCount: 1; sex: male; lifeStage: adult; **Taxon:** kingdom: Animalia; phylum: Arthropoda; class: Insecta; order: Diptera; family: Phoridae; genus: Megaselia; specificEpithet: friedrichae; scientificNameAuthorship: Hartop, Brown, & Disney; **Location:** country: USA; stateProvince: California; municipality: Los Angeles; locality: Silver Lake; **Event:** samplingProtocol: Malaise trap; verbatimEventDate: 28.VI-5.VII.2014; **Record Level:** institutionCode: LACM; collectionCode: ENT
- **Type status:** Paratype. **Occurrence:** catalogNumber: 322025, 322026; recordedBy: Hogg; individualCount: 2; sex: male; lifeStage: adult; **Taxon:** kingdom: Animalia; phylum: Arthropoda; class: Insecta; order: Diptera; family: Phoridae; genus: Megaselia; specificEpithet: friedrichae; scientificNameAuthorship: Hartop, Brown, & Disney; **Location:** country: USA; stateProvince: California; municipality: Los Angeles; locality: Silver Lake; **Event:** samplingProtocol: Malaise trap; verbatimEventDate: 2-9.VIII.2014; **Record Level:** institutionCode: LACM; collectionCode: ENT
- **Type status:** Paratype. **Occurrence:** recordedBy: Hogg; individualCount: 2; sex: male; lifeStage: adult; **Taxon:** kingdom: Animalia; phylum: Arthropoda; class: Insecta; order: Diptera; family: Phoridae; genus: Megaselia; specificEpithet: friedrichae; scientificNameAuthorship: Hartop, Brown, & Disney; **Location:** country: USA; stateProvince: California; municipality: Los Angeles; locality: Silver Lake; **Event:** samplingProtocol: Malaise trap; verbatimEventDate: 28.VI-5.VII.2014; **Record Level:** institutionCode: CUMZ
- **Type status:** Other material. **Occurrence:** recordedBy: Creason; individualCount: 4; sex: male; lifeStage: adult; **Taxon:** kingdom: Animalia; phylum: Arthropoda; class: Insecta; order: Diptera; family: Phoridae; genus: Megaselia; specificEpithet: friedrichae; scientificNameAuthorship: Hartop, Brown, & Disney; **Location:** country: USA; stateProvince: California; municipality: Los Angeles; locality: Glassell Park; **Event:** samplingProtocol: Malaise trap; verbatimEventDate: V-VIII.2014; **Record Level:** institutionCode: LACM; collectionCode: ENT

#### Description

**Figure 2.**
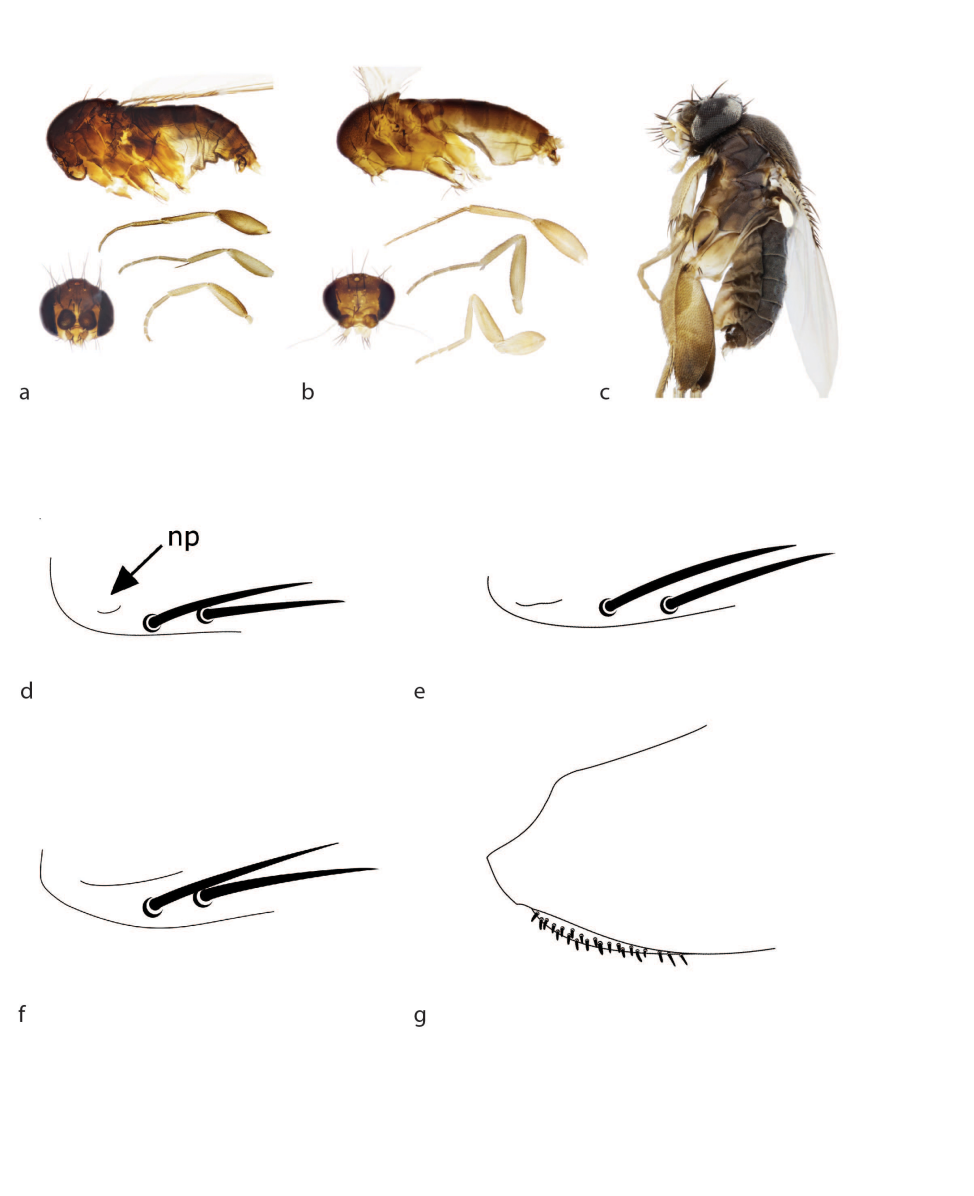
Habitus images (left lateral). a. *Megaselia studentorum*. b. *Megaselia voluntariorum*. c. *Megaselia wongae*. Notopleural cleft (np). d. *Megaselia friedrichae*. e. *Megaselia losangelensis*. f. *Megaselia pongsaiae*. Ventral side of base of left hind femur. g. *Megaselia stoakesi*.

**Figure 3.**
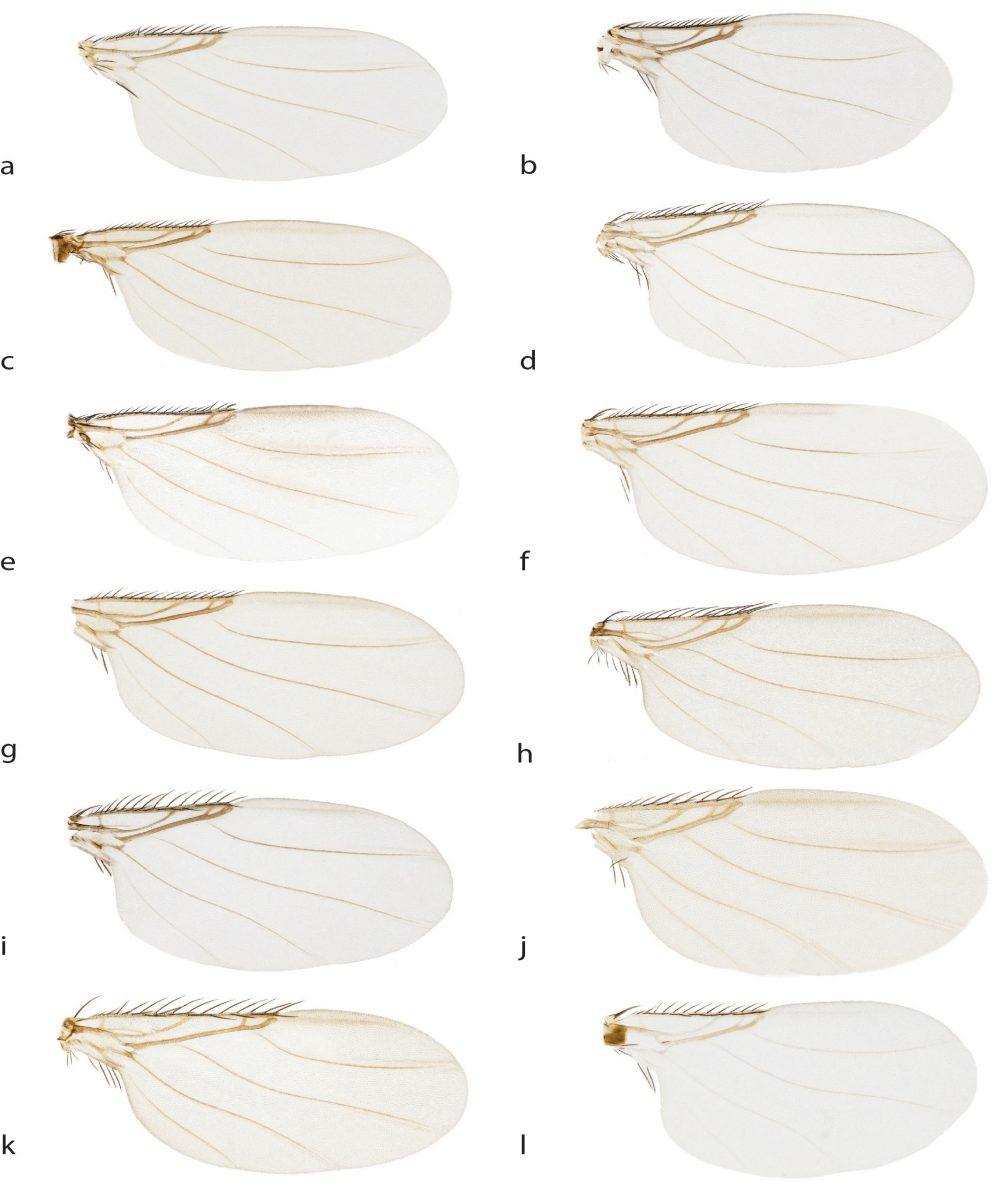
Right wing, dorsal. a. *Megaselia baileyae*. b. *Megaselia friedrichae*. c. *Megaselia gonzalezorum*. d. *Megaselia joanneae*. e. *Megaselia losangelensis*. f. *Megaselia phyllissunae*. g. *Megaselia pongsaiae*. h. *Megaselia shatesae*. i. *Megaselia stoakesi*. j. *Megaselia studentorum*. k. *Megaselia voluntariorum*. l. *Megaselia wongae*.

**Figure 4.**
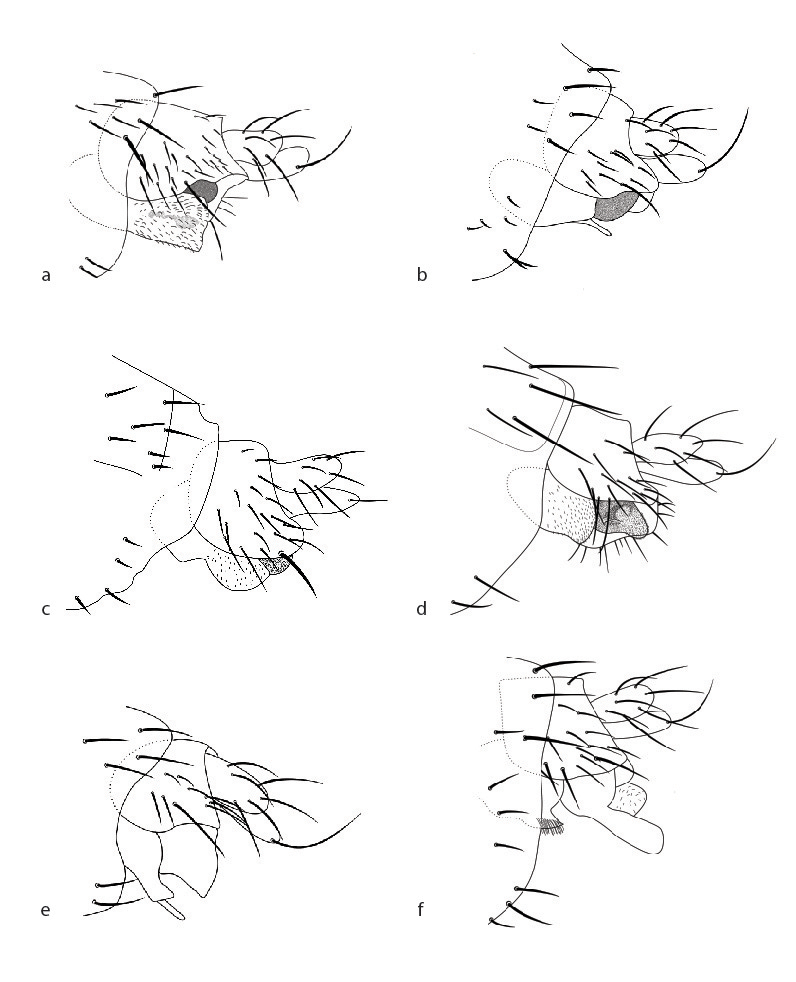
Male genitalia, left lateral. a. *Megaselia baileyae*. b. *Megaselia friedrichae*. c. *Megaselia gonzalezorum*. d. *Megaselia joanneae*. e. *Megaselia losangelensis*. f. *Megaselia phyllissunae*.

See description Table 1 and Fig. 1b, Fig. 2d, Fig. 3b, Fig. 4b.

#### Diagnosis

Male. In the group VIII key of Borgmeier (1966), *M. friedrichae* keys to couplet 9 where it differs from *M. berndseni* by the presence of a notopleural cleft and cannot be taken further in the key due to its short costal index (0.34-0.35). If one assumes a margin of error and takes *M. friedrichae* further in the key despite the short costal index, at couplet 11 it differs from *M. globipyga* by having a notopleural cleft and from *M. brevicostalis* by having a very small notopleural cleft (Fig. 2d) versus the large cleft on *M. brevicostalis*.

#### Etymology

Named by BioSCAN Phase I Project Manager Dean Pentcheff in honor of Kristin Friedrich whose effective work on behalf of this project brought a wide audience into an appreciation of the richness of urban biodiversity.

#### Distribution

Los Angeles, California (USA).

#### Biology

Unknown.

### Megaselia gonzalezorum

Hartop, Brown, & Disney 2016 sp. n.

urn:lsid:zoobank.org:act:05302326-570E-4575-A91B-846F41CE68FE

#### Materials

- **Type status:** Holotype. **Occurrence:** catalogNumber: 322019; recordedBy: Oxborough; individualCount: 1; sex: male; lifeStage: adult; **Taxon:** kingdom: Animalia; phylum: Arthropoda; class: Insecta; order: Diptera; family: Phoridae; genus: Megaselia; specificEpithet: gonzalezorum; scientificNameAuthorship: Hartop, Brown, & Disney; **Location:** country: USA; stateProvince: California; municipality: Los Angeles; locality: Mid-City; **Event:** samplingProtocol: Malaise trap; verbatimEventDate: 1-8.XI.2014; **Record Level:** institutionCode: LACM; collectionCode: ENT
- **Type status:** Paratype. **Occurrence:** catalogNumber: 322020; recordedBy: Keller; individualCount: 1; sex: male; lifeStage: adult; **Taxon:** kingdom: Animalia; phylum: Arthropoda; class: Insecta; order: Diptera; family: Phoridae; genus: Megaselia; specificEpithet: gonzalezorum; scientificNameAuthorship: Hartop, Brown, & Disney; **Location:** country: USA; stateProvince: California; municipality: Los Angeles; locality: Eagle Rock; **Event:** samplingProtocol: Malaise trap; verbatimEventDate: 1-8.II.2014; **Record Level:** institutionCode: LACM; collectionCode: ENT
- **Type status:** Paratype. **Occurrence:** catalogNumber: 322073; recordedBy: Donahue; individualCount: 1; sex: male; lifeStage: adult; **Taxon:** kingdom: Animalia; phylum: Arthropoda; class: Insecta; order: Diptera; family: Phoridae; genus: Megaselia; specificEpithet: gonzalezorum; scientificNameAuthorship: Hartop, Brown, & Disney; **Location:** country: USA; stateProvince: California; municipality: Los Angeles; locality: Mount Washington; **Event:** samplingProtocol: Malaise trap; verbatimEventDate: 1-8.III.2014; **Record Level:** institutionCode: LACM; collectionCode: ENT
- **Type status:** Other material. **Occurrence:** occurrenceRemarks: specimen badly damaged; recordedBy: Marquez; individualCount: 1; sex: male; lifeStage: adult; **Taxon:** kingdom: Animalia; phylum: Arthropoda; class: Insecta; order: Diptera; family: Phoridae; genus: Megaselia; specificEpithet: gonzalezorum; scientificNameAuthorship: Hartop, Brown, & Disney; **Location:** country: USA; stateProvince: California; municipality: Los Angeles; locality: Pico-Union; **Event:** samplingProtocol: Malaise trap; verbatimEventDate: XI.2014; **Record Level:** institutionCode: LACM; collectionCode: ENT

#### Description

See description Table 1 and Fig. 1c, Fig. 3c, Fig. 4c.

#### Diagnosis

Male. In the group VIII key of Borgmeier (1966), *M. gonzalezorum* keys to couplet nine, where its costal index of 0.35-0.36 differentiates it from either the very short (0.33) costal index of *M. pygmaeoides* [now considered to be *M. berndseni* (Schmitz 1919)] or the longer costal index (0.38-0.43) for continuing in the key. It also differs from *M. berndseni* in details of the genitalia (Fig. 4c), and if taken further in the key (by assuming that with more specimens the costal index might sometimes reach 0.38), details of the genitalia including the "nose-like" shape of the epandrium, would quickly differentiate it from species listed further down.

#### Etymology

Named by BioSCAN employee Lisa Gonzalez in honor of her family; parents Armando and Aida Gonzalez, and sister Rita Gonzalez.

#### Distribution

Los Angeles, California (USA).

#### Biology

Unknown.

### Megaselia joanneae

Hartop, Brown, & Disney 2016 sp. n.

urn:lsid:zoobank.org:act:F4CB4149-409B-44C0-9B04-294C53E5107D

#### Materials

- **Type status:** Holotype. **Occurrence:** catalogNumber: 322018; recordedBy: Brown; individualCount: 1; sex: male; lifeStage: adult; **Taxon:** kingdom: Animalia; phylum: Arthropoda; class: Insecta; order: Diptera; family: Phoridae; genus: Megaselia; specificEpithet: joanneae; scientificNameAuthorship: Hartop, Brown, & Disney; **Location:** country: USA; stateProvince: California; municipality: Los Angeles; locality: Monrovia; **Event:** samplingProtocol: Malaise trap; verbatimEventDate: 30.I-6.II.2011; **Record Level:** institutionCode: LACM; collectionCode: ENT
- **Type status:** Paratype. **Occurrence:** catalogNumber: 322016; recordedBy: Hogg; individualCount: 1; sex: male; lifeStage: adult; **Taxon:** kingdom: Animalia; phylum: Arthropoda; class: Insecta; order: Diptera; family: Phoridae; genus: Megaselia; specificEpithet: joanneae; scientificNameAuthorship: Hartop, Brown, & Disney; **Location:** country: USA; stateProvince: California; municipality: Los Angeles; locality: Silver Lake; **Event:** samplingProtocol: Malaise trap; verbatimEventDate: 1-8.II.2014; **Record Level:** institutionCode: LACM; collectionCode: ENT
- **Type status:** Paratype. **Occurrence:** catalogNumber: 322017; recordedBy: Oxborough; individualCount: 1; sex: male; lifeStage: adult; **Taxon:** kingdom: Animalia; phylum: Arthropoda; class: Insecta; order: Diptera; family: Phoridae; genus: Megaselia; specificEpithet: joanneae; scientificNameAuthorship: Hartop, Brown, & Disney; **Location:** country: USA; stateProvince: California; municipality: Los Angeles; locality: Mid-City; **Event:** samplingProtocol: Malaise trap; verbatimEventDate: 28.XII.2013-4.I.2014; **Record Level:** institutionCode: LACM; collectionCode: ENT
- **Type status:** Paratype. **Occurrence:** recordedBy: Harding; individualCount: 1; sex: male; lifeStage: adult; **Taxon:** kingdom: Animalia; phylum: Arthropoda; class: Insecta; order: Diptera; family: Phoridae; genus: Megaselia; specificEpithet: joanneae; scientificNameAuthorship: Hartop, Brown, & Disney; **Location:** country: USA; stateProvince: California; municipality: Los Angeles; locality: Elysian Park; **Event:** samplingProtocol: Malaise trap; verbatimEventDate: 28.XII.2013-4.I.2014; **Record Level:** institutionCode: CUMZ
- **Type status:** Other material. **Occurrence:** occurrenceRemarks: former M. postcrinata paratype; recordedBy: Dewatto; individualCount: 1; sex: male; lifeStage: adult; **Taxon:** kingdom: Animalia; phylum: Arthropoda; class: Insecta; order: Diptera; family: Phoridae; genus: Megaselia; specificEpithet: joanneae; scientificNameAuthorship: Hartop, Brown, & Disney; **Location:** country: USA; stateProvince: Oregon; municipality: Portland; **Event:** samplingProtocol: Malaise trap; verbatimEventDate: 20.VII.1909; **Record Level:** institutionCode: USNM
- **Type status:** Other material. **Occurrence:** occurrenceRemarks: former M. postcrinata paratype; recordedBy: Melander; individualCount: 1; sex: male; lifeStage: adult; **Taxon:** kingdom: Animalia; phylum: Arthropoda; class: Insecta; order: Diptera; family: Phoridae; genus: Megaselia; specificEpithet: joanneae; scientificNameAuthorship: Hartop, Brown, & Disney; **Location:** country: USA; stateProvince: Washington; municipality: Mukilteo; **Event:** samplingProtocol: Malaise trap; verbatimEventDate: 1.VII.1924; **Record Level:** institutionCode: USNM
- **Type status:** Other material. **Occurrence:** occurrenceRemarks: former M. postcrinata paratype; individualCount: 1; sex: male; lifeStage: adult; **Taxon:** kingdom: Animalia; phylum: Arthropoda; class: Insecta; order: Diptera; family: Phoridae; genus: Megaselia; specificEpithet: joanneae; scientificNameAuthorship: Hartop, Brown, & Disney; **Location:** country: USA; stateProvince: Washington; municipality: Dewatto; **Event:** samplingProtocol: Malaise trap; verbatimEventDate: 15.VIII.1910; **Record Level:** institutionCode: USNM

#### Description

See description Table 1 and Fig. 1d, Fig. 3d, Fig. 4d.

#### Diagnosis

Male. In the group VIII key of Borgmeier (1966), *M. joanneae* keys to couplet 11 where it differs from *M. globipyga* Borgmeier by lacking the globose genitalia of that species and from *M. brevicostalis* Wood 1910 by having unequal supraantennals and lacking the large notopleural cleft of that species. It should be noted that in order to key to *M. globipyga*, you must say that the first costal division is at most as long as second (couplet 10), which is not always accurate (but the alternative, of the first costal division being three-times as long as second, is much less accurate). Otherwise, the species simply fails to key at couplet 10. Within the Los Angeles fauna, this species is quite similar to *M. mikejohnsoni*, but differs in details of the genitalia, most noticeably with *M. joanneae* lacking the prominent trio of setae on the posterior of the epandrium (Fig. 83 in Hartop et al. 2015 versus Fig. 4d).

#### Etymology

Named by Adam and Jenessa Wall in honor of their mother, JoAnne Kay Wall.

#### Distribution

Los Angeles, California (USA).

#### Biology

Unknown.

### Megaselia losangelensis

Hartop, Brown, & Disney 2016 sp. n.

urn:lsid:zoobank.org:act:4BFC18EA-8CF8-4430-A3D9-D9368CFC1C42

#### Materials

- **Type status:** Holotype. **Occurrence:** catalogNumber: 322027; recordedBy: Hogg; individualCount: 1; sex: male; lifeStage: adult; **Taxon:** kingdom: Animalia; phylum: Arthropoda; class: Insecta; order: Diptera; family: Phoridae; genus: Megaselia; specificEpithet: losangelensis; scientificNameAuthorship: Hartop, Brown, & Disney; **Location:** country: USA; stateProvince: California; municipality: Los Angeles; locality: Silver Lake; **Event:** samplingProtocol: Malaise trap; verbatimEventDate: 26.IV-10.V.2014; **Record Level:** institutionCode: LACM; collectionCode: ENT
- **Type status:** Paratype. **Occurrence:** catalogNumber: 322028; recordedBy: Armstrong; individualCount: 1; sex: male; lifeStage: adult; **Taxon:** kingdom: Animalia; phylum: Arthropoda; class: Insecta; order: Diptera; family: Phoridae; genus: Megaselia; specificEpithet: losangelensis; scientificNameAuthorship: Hartop, Brown, & Disney; **Location:** country: USA; stateProvince: California; municipality: Los Angeles; locality: Glendale; **Event:** samplingProtocol: Malaise trap; verbatimEventDate: 28.VI-5.VII.2014; **Record Level:** institutionCode: LACM; collectionCode: ENT
- **Type status:** Paratype. **Occurrence:** catalogNumber: 322029; recordedBy: Keller; individualCount: 1; sex: male; lifeStage: adult; **Taxon:** kingdom: Animalia; phylum: Arthropoda; class: Insecta; order: Diptera; family: Phoridae; genus: Megaselia; specificEpithet: losangelensis; scientificNameAuthorship: Hartop, Brown, & Disney; **Location:** country: USA; stateProvince: California; municipality: Los Angeles; locality: Eagle Rock; **Event:** samplingProtocol: Malaise trap; verbatimEventDate: 2-19.VIII.2014; **Record Level:** institutionCode: LACM; collectionCode: ENT
- **Type status:** Paratype. **Occurrence:** recordedBy: Dahl; individualCount: 1; sex: male; lifeStage: adult; **Taxon:** kingdom: Animalia; phylum: Arthropoda; class: Insecta; order: Diptera; family: Phoridae; genus: Megaselia; specificEpithet: losangelensis; scientificNameAuthorship: Hartop, Brown, & Disney; **Location:** country: USA; stateProvince: California; municipality: Los Angeles; locality: Carthay; **Event:** samplingProtocol: Malaise trap; verbatimEventDate: 29.VII-5.VIII.2014; **Record Level:** institutionCode: CUMZ
- **Type status:** Paratype. **Occurrence:** recordedBy: Higgins; individualCount: 1; sex: male; lifeStage: adult; **Taxon:** kingdom: Animalia; phylum: Arthropoda; class: Insecta; order: Diptera; family: Phoridae; genus: Megaselia; specificEpithet: losangelensis; scientificNameAuthorship: Hartop, Brown, & Disney; **Location:** country: USA; stateProvince: Oregon; municipality: Los Angeles; locality: Atwater Village; **Event:** samplingProtocol: Malaise trap; verbatimEventDate: 26.XI-10.XII.2014; **Record Level:** institutionCode: CUMZ

#### Description

See description Table 1 and Fig. 2e, Fig. 3e, Fig. 4e.

#### Diagnosis

Male. In the group VIII key of Borgmeier (1966), *M. losangelensis* keys to couplet 10 where it fails the key by having costal division one greater than two-times, but not as long as three times, costal division two. Assuming a margin of error, if one continues to couplet 11 *M. losangelensis* differs from *M. globipyga* by having a cleft, and from *M. brevicostalis* by having a small cleft (Fig. 2e) as opposed to the large cleft on *M. brevicostalis*.

#### Etymology

Named by BioSCAN Phase I Co-Principal Investigator Regina Wetzer in honor of our “City of Angels”, the place that made BioSCAN possible.

#### Distribution

Los Angeles, California (USA).

#### Biology

Unknown.

### Megaselia phyllissunae

Hartop, Brown, & Disney 2016 sp. n.

urn:lsid:zoobank.org:act:BE98338B-CB4E-4B1D-8DF7-F10BCA3E78F0

#### Materials

- **Type status:** Holotype. **Occurrence:** catalogNumber: 322041; recordedBy: Brown; individualCount: 1; sex: male; lifeStage: adult; **Taxon:** kingdom: Animalia; phylum: Arthropoda; class: Insecta; order: Diptera; family: Phoridae; genus: Megaselia; specificEpithet: phyllissunae; scientificNameAuthorship: Hartop, Brown, & Disney; **Location:** country: USA; stateProvince: California; municipality: Los Angeles; locality: Monrovia; **Event:** samplingProtocol: Malaise trap; verbatimEventDate: 30.I-6.II.2011; **Record Level:** institutionCode: LACM; collectionCode: ENT
- **Type status:** Paratype. **Occurrence:** catalogNumber: 322042; recordedBy: Brejcha; individualCount: 1; sex: male; lifeStage: adult; **Taxon:** kingdom: Animalia; phylum: Arthropoda; class: Insecta; order: Diptera; family: Phoridae; genus: Megaselia; specificEpithet: phyllissunae; scientificNameAuthorship: Hartop, Brown, & Disney; **Location:** country: USA; stateProvince: California; municipality: Los Angeles; locality: Highland Park; **Event:** samplingProtocol: Malaise trap; verbatimEventDate: 1-8.II.2014; **Record Level:** institutionCode: LACM; collectionCode: ENT
- **Type status:** Paratype. **Occurrence:** catalogNumber: 322043; recordedBy: Hogue; individualCount: 1; sex: male; lifeStage: adult; **Taxon:** kingdom: Animalia; phylum: Arthropoda; class: Insecta; order: Diptera; family: Phoridae; genus: Megaselia; specificEpithet: phyllissunae; scientificNameAuthorship: Hartop, Brown, & Disney; **Location:** country: USA; stateProvince: California; municipality: Los Angeles; locality: Eagle Rock; **Event:** samplingProtocol: Malaise trap; verbatimEventDate: 1-8.III.2014; **Record Level:** institutionCode: LACM; collectionCode: ENT

#### Description

See description Table 1 and Fig. 1f, Fig. 3f, Fig. 4f.

#### Diagnosis

Male. In the group VIII key of Borgmeier (1966), *M. phyllissunae* keys to couplet 11 where it differs from *M. globipyga* Borgmeier by lacking the globose genitalia of that species (Fig. 3 in Hartop et al. 2016 versus Fig. 4f) and from *M. brevicostalis* Wood by having unequal supraantennals and lacking the notopleural cleft of that species.

#### Etymology

Named in honor of Phyllis Sun for her many contributions to phase I of the BioSCAN project.

#### Distribution

Los Angeles, California (USA).

#### Biology

Unknown.

### Megaselia pongsaiae

Hartop, Brown, & Disney 2016 sp. n.

urn:lsid:zoobank.org:act:97A19D0B-6429-43C5-B7C9-3E2C66F4C9AD

#### Materials

- **Type status:** Holotype. **Occurrence:** catalogNumber: 322030; recordedBy: Dahl; individualCount: 1; sex: male; lifeStage: adult; **Taxon:** kingdom: Animalia; phylum: Arthropoda; class: Insecta; order: Diptera; family: Phoridae; genus: Megaselia; specificEpithet: pongsaiae; scientificNameAuthorship: Hartop, Brown, & Disney; **Location:** country: USA; stateProvince: California; municipality: Los Angeles; locality: Carthay; **Event:** samplingProtocol: Malaise trap; verbatimEventDate: 29.VII-5.VIII.2014; **Record Level:** institutionCode: LACM; collectionCode: ENT
- **Type status:** Paratype. **Occurrence:** catalogNumber: 322031; recordedBy: Brejcha; individualCount: 1; sex: male; lifeStage: adult; **Taxon:** kingdom: Animalia; phylum: Arthropoda; class: Insecta; order: Diptera; family: Phoridae; genus: Megaselia; specificEpithet: pongsaiae; scientificNameAuthorship: Hartop, Brown, & Disney; **Location:** country: USA; stateProvince: California; municipality: Los Angeles; locality: Highland Park; **Event:** samplingProtocol: Malaise trap; verbatimEventDate: 1-8.II.2014; **Record Level:** institutionCode: LACM; collectionCode: ENT

#### Description

**Figure 5.**
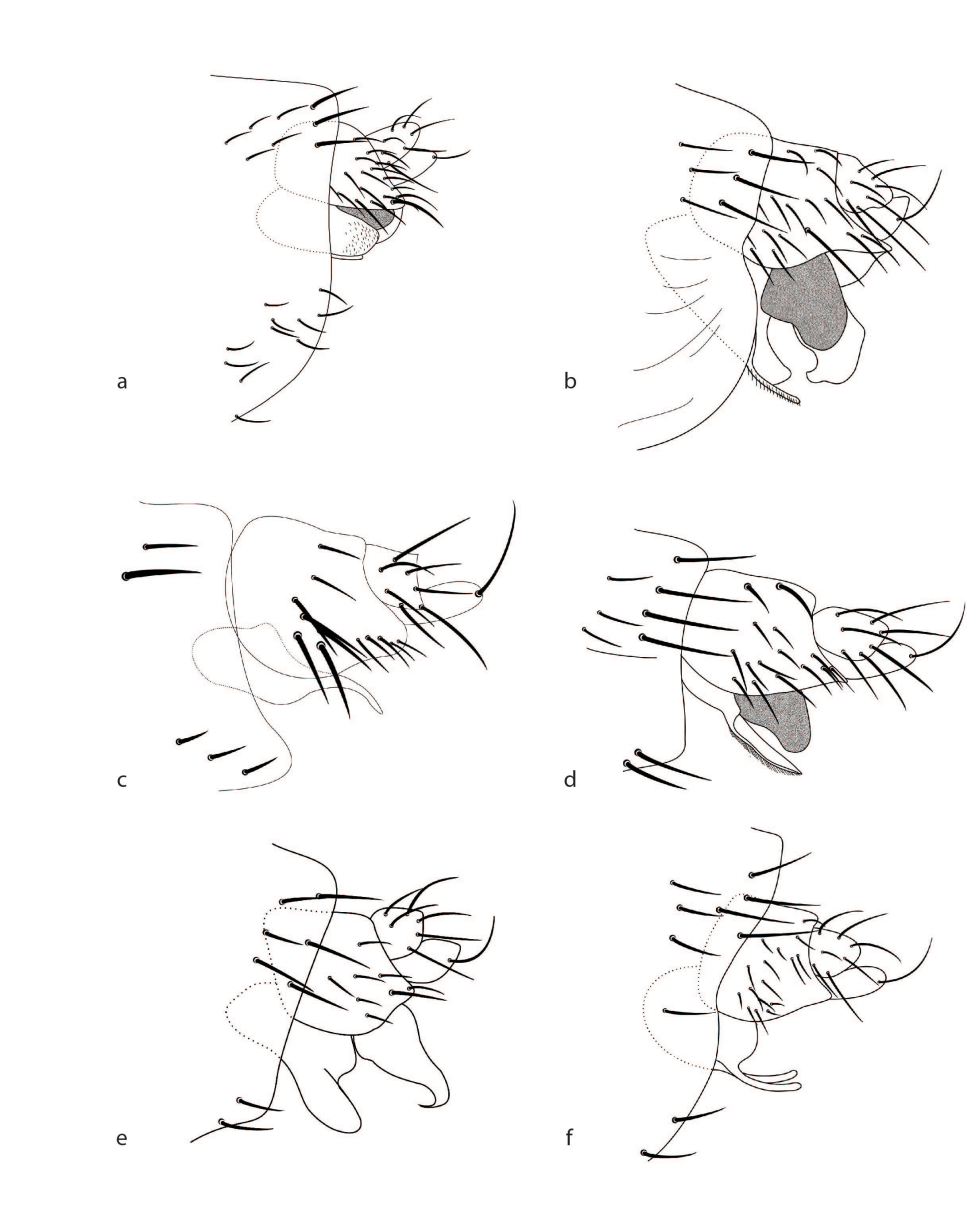
Male genitalia, left lateral. a. *Megaselia pongsaiae*. b. *Megaselia shatesae*. c. *Megaselia stoakesi*. d. *Megaselia studentorum*. e. *Megaselia voluntariorum*. f. *Megaselia wongae*.

See description Table 2 and Fig. 1g, Fig. 2f, Fig. 3g, Fig. 5a.

#### Diagnosis

Male. In the group VIII key of Borgmeier (1966), *M. pongsaiae* keys to couplet 19 where it keys to *M. polyporicola* Borgmeier. These species differ in a number of ways, but most easily *M. pongsaiae* has a long seta at the base of vein R on the wing, and *M. polyporicola* lacks a seta in this position.

#### Etymology

Named by Kathy Omura in honor of USC work study student Jean Pongsai for her hard work training and supervising students.

#### Distribution

Los Angeles, California (USA).

#### Biology

Unknown.

### Megaselia shatesae

Hartop, Brown, & Disney 2016 sp. n.

urn:lsid:zoobank.org:act:04CC1F03-E220-4ADB-96F5-79D110676096

#### Materials

- **Type status:** Holotype. **Occurrence:** catalogNumber: 322021; recordedBy: Dahl; individualCount: 1; sex: male; lifeStage: adult; **Taxon:** kingdom: Animalia; phylum: Arthropoda; class: Insecta; order: Diptera; family: Phoridae; genus: Megaselia; specificEpithet: shatesae; scientificNameAuthorship: Hartop, Brown, & Disney; **Location:** country: USA; stateProvince: California; municipality: Los Angeles; locality: Carthay; **Event:** samplingProtocol: Malaise trap; verbatimEventDate: 2-9.XII.2014; **Record Level:** institutionCode: LACM; collectionCode: ENT
- **Type status:** Paratype. **Occurrence:** catalogNumber: 322022; recordedBy: Hentschke; individualCount: 1; sex: male; lifeStage: adult; **Taxon:** kingdom: Animalia; phylum: Arthropoda; class: Insecta; order: Diptera; family: Phoridae; genus: Megaselia; specificEpithet: shatesae; scientificNameAuthorship: Hartop, Brown, & Disney; **Location:** country: USA; stateProvince: California; municipality: Los Angeles; locality: University Park; **Event:** samplingProtocol: Malaise trap; verbatimEventDate: 3-10.XII.2014; **Record Level:** institutionCode: LACM; collectionCode: ENT

#### Description

See description Table 2 and Fig. 1h, Fig. 3h, Fig. 5b.

#### Diagnosis

Male. In the group VIII key of Borgmeier (1966), *M. shatesae* keys to couplet 19 where it differs from both *M. perplexa* Malloch and *M. polyporicola* Borgmeier by its very long costal setae (0.18 mm). This species is superficially similar to some other species found in Los Angeles, but the combination of dark halters, very long costal setae, 5 alular setae, and a strong, complete subcosta make this species easily separated from any potential lookalike.

#### Etymology

Named in honor of Tessa Shates whose volunteer work with author Hartop helped in identifying these twelve new species.

#### Distribution

Los Angeles, California (USA).

#### Biology

Unknown.

### Megaselia stoakesi

Hartop, Brown, & Disney 2016 sp. n.

urn:lsid:zoobank.org:act:3782229F-CC58-4372-97F5-ADDA2C0D9325

#### Materials

- **Type status:** Holotype. **Occurrence:** catalogNumber: 9-12; recordedBy: Ralph Stoakes; individualCount: 1; sex: male; lifeStage: adult; **Taxon:** kingdom: Animalia; phylum: Arthropoda; class: Insecta; order: Diptera; family: Phoridae; genus: Megaselia; specificEpithet: stoakesi; scientificNameAuthorship: Hartop, Brown, & Disney; **Location:** country: USA; stateProvince: Colorado; county: Larimer; verbatimLocality: Soapstone Prairies Natural Area, Spittlewood Creek; **Event:** samplingProtocol: Malaise trap; verbatimEventDate: 10.X.2010; **Record Level:** institutionCode: CUMZ
- **Type status:** Paratype. **Occurrence:** catalogNumber: 322009; recordedBy: Donahue; individualCount: 1; sex: male; lifeStage: adult; **Taxon:** kingdom: Animalia; phylum: Arthropoda; class: Insecta; order: Diptera; family: Phoridae; genus: Megaselia; specificEpithet: stoakesi; scientificNameAuthorship: Hartop, Brown, & Disney; **Location:** country: USA; stateProvince: California; municipality: Los Angeles; locality: Mount Washington; **Event:** samplingProtocol: Malaise trap; verbatimEventDate: 1-8.XI.2014; **Record Level:** institutionCode: LACM; collectionCode: ENT

#### Description

See description Table 2 and Fig. 1i, Fig. 2g, Fig. 3i, Fig. 5c.

#### Diagnosis

Male. In the keys of Borgmeier (1964)​, this species can be taken through either the group III or group V key, depending on whether one takes the stronger setae on the posterior edge of the anepisternum as strong hairs or weak bristles. In the key to group III, it keys to couplets 13 or 14 (depending on whether the setation on the epandrium is judged to be bristles or bristlelike hairs), and in the key to group V it keys to couplets 6 or 7 (depending on whether the brown palps are considered to be black or yellow). It can easily be differentiated from any of the species found at these couplets by the presence of dense fields of short, blunt spines on f3 basally (Fig. 2g).

#### Etymology

Named in honor of Ralph Stoakes, the collector of the holotype.

#### Distribution

Los Angeles, California and Larimer County, Colorado (USA).

#### Biology

Unknown.

### Megaselia studentorum

Hartop, Brown, & Disney 2016 sp. n.

urn:lsid:zoobank.org:act:C0EA59C0-D1E0-4D17-B960-718384FE4CA9

#### Materials

- **Type status:** Holotype. **Occurrence:** catalogNumber: 322039; recordedBy: Johnson; individualCount: 1; sex: male; lifeStage: adult; **Taxon:** kingdom: Animalia; phylum: Arthropoda; class: Insecta; order: Diptera; family: Phoridae; genus: Megaselia; specificEpithet: studentorum; scientificNameAuthorship: Hartop, Brown, & Disney; **Location:** country: USA; stateProvince: California; municipality: Los Angeles; locality: Echo Park; **Event:** samplingProtocol: Malaise trap; verbatimEventDate: 3-10.VI.2014; **Record Level:** institutionCode: LACM; collectionCode: ENT

#### Description

See description Table 2 and Fig. 2a, Fig. 3j, Fig. 5d.

#### Diagnosis

Male. In the group VII key of Borgmeier (1966), *M. studentorum* keys to *M. inornata* Malloch at couplet 41. *Megaselia studentorum* differs from both *M. inornata* and most others species by its costal ratio of 2.40: 1.85: 1; costal segment 2 is longer relative to the other two segments than is usual. *Megaselia inornata*, for instance, has a ratio of 2.78: 1.44: 1 (25: 13: 9), this is more typical in *Megaselia*.

#### Etymology

Named in honor of the many USC work study students who continue to do countless hours of work on every aspect of the BioSCAN project. Without them, BioSCAN would not be possible.

#### Distribution

Los Angeles, California (USA).

#### Biology

Unknown.

### Megaselia voluntariorum

Hartop, Brown, & Disney 2016 sp. n.

urn:lsid:zoobank.org:act:FD866146-AA4D-4BE9-8490-09ED7BD87C00

#### Materials

- **Type status:** Holotype. **Occurrence:** catalogNumber: 322040; recordedBy: Hoffman; individualCount: 1; sex: male; lifeStage: adult; **Taxon:** kingdom: Animalia; phylum: Arthropoda; class: Insecta; order: Diptera; family: Phoridae; genus: Megaselia; specificEpithet: voluntariorum; scientificNameAuthorship: Hartop, Brown, & Disney; **Location:** country: USA; stateProvince: California; municipality: Los Angeles; locality: Glendale; **Event:** samplingProtocol: Malaise trap; verbatimEventDate: 31.V-7.VI.2014; **Record Level:** institutionCode: LACM; collectionCode: ENT

#### Description

See description Table 2 and Fig. 2b, Fig. 3k, Fig. 5e.

#### Diagnosis

Male. In the group VIII key of Borgmeier (1966), *M. voluntariorum* keys to couplet 19. At this couplet, *M. perplexa* Malloch is described only from the female and thus ruled out, and *M. polyporicola* lacks a seta at the base of R on the wing, while *M. voluntariorum* has a short seta. Compared to other *Megaselia* from the Los Angeles area with a complete subcosta, *M. voluntariorum* has a short wing (just over 1mm) and short costal index (0.38).

#### Etymology

Named in honor of the many people that have, are, and will volunteer for the BioSCAN project. These volunteers are critical to our operation, and have contributed to everything from public outreach in the NHM Nature Lab to specialized work on phorid flies.

#### Distribution

Los Angeles, California (USA).

#### Biology

Unknown.

### Megaselia wongae

Hartop, Brown, & Disney 2016 sp. n.

urn:lsid:zoobank.org:act:E1CA6A5E-4297-4D93-863B-C124BBFA59CD

#### Materials

- **Type status:** Holotype. **Occurrence:** catalogNumber: 322010; recordedBy: Harding; individualCount: 1; sex: male; lifeStage: adult; **Taxon:** kingdom: Animalia; phylum: Arthropoda; class: Insecta; order: Diptera; family: Phoridae; genus: Megaselia; specificEpithet: wongae; scientificNameAuthorship: Hartop, Brown, & Disney; **Location:** country: USA; stateProvince: California; municipality: Los Angeles; locality: Elysian Park; **Event:** samplingProtocol: Malaise trap; verbatimEventDate: 30.VIII-6.IX.2014; **Record Level:** institutionCode: LACM; collectionCode: ENT
- **Type status:** Paratype. **Occurrence:** catalogNumber: 322011; recordedBy: Keller; individualCount: 1; sex: male; lifeStage: adult; **Taxon:** kingdom: Animalia; phylum: Arthropoda; class: Insecta; order: Diptera; family: Phoridae; genus: Megaselia; specificEpithet: wongae; scientificNameAuthorship: Hartop, Brown, & Disney; **Location:** country: USA; stateProvince: California; municipality: Los Angeles; locality: Eagle Rock; **Event:** samplingProtocol: Malaise trap; verbatimEventDate: 27.IX-4.X.2014; **Record Level:** institutionCode: LACM; collectionCode: ENT
- **Type status:** Paratype. **Occurrence:** catalogNumber: 322012; recordedBy: Brejcha; individualCount: 1; sex: male; lifeStage: adult; **Taxon:** kingdom: Animalia; phylum: Arthropoda; class: Insecta; order: Diptera; family: Phoridae; genus: Megaselia; specificEpithet: wongae; scientificNameAuthorship: Hartop, Brown, & Disney; **Location:** country: USA; stateProvince: California; municipality: Los Angeles; locality: Highland Park; **Event:** samplingProtocol: Malaise trap; verbatimEventDate: 25.X-8.XI.2014; **Record Level:** institutionCode: LACM; collectionCode: ENT
- **Type status:** Paratype. **Occurrence:** recordedBy: Brejcha; individualCount: 2; sex: male; lifeStage: adult; **Taxon:** kingdom: Animalia; phylum: Arthropoda; class: Insecta; order: Diptera; family: Phoridae; genus: Megaselia; specificEpithet: wongae; scientificNameAuthorship: Hartop, Brown, & Disney; **Location:** country: USA; stateProvince: California; municipality: Los Angeles; locality: Highland Park; **Event:** samplingProtocol: Malaise trap; verbatimEventDate: 25.X-8.XI.2014; **Record Level:** institutionCode: CUMZ
- **Type status:** Other material. **Occurrence:** individualCount: 32; sex: male; lifeStage: adult; **Taxon:** kingdom: Animalia; phylum: Arthropoda; class: Insecta; order: Diptera; family: Phoridae; genus: Megaselia; specificEpithet: wongae; scientificNameAuthorship: Hartop, Brown, & Disney; **Location:** country: USA; stateProvince: California; municipality: Los Angeles; locality: Gardena, Highland Park, Mount Washington, Glendale, Eagle Rock, Atwater Village, Los Feliz, Elysian Park, Silver Lake; **Event:** samplingProtocol: Malaise trap; verbatimEventDate: I-XII.2014; **Record Level:** institutionCode: LACM; collectionCode: ENT

#### Description

See description Table 2 and Fig. 2c, Fig. 3l, Fig. 5f.

#### Diagnosis

Male. In the group VIII key of Borgmeier (1966), *M. wongae* keys to *M. bovista* [now considered to be *M. agarici* (Lintner, 1895)], from which it can easily be differentiated by the absence of *M. agarici’s* characteristic pale protrusion from the posterior of the epandrium. This species is similar to *M. lombardorum* Hartop et al. (2015), also from the L.A. region, but differs in having much longer setae on T6, and hypandrial lobes that appeared cupped (Hartop et al. 2015: fig 81 versus Fig. 5f).

#### Etymology

Named in honor of BioSCAN volunteer Maria Wong whose help identifying *Megaselia* for BioSCAN Phase I contributed to the discovery of these twelve new species.

#### Distribution

Los Angeles, California (USA).

#### Biology

Unknown.

## Discussion

The authors continue to stress that the field of species-level taxonomy, especially in urban environments, must continue to grow. Taxonomists and their funding agencies must give time, attention and money to the environments surrounding their towns and cities. Poor quality type material continues to be an obstacle to identification of *Megaselia*. Dissecting and slide mounting old type specimens has proven to be the only method for any sort of definitive identification. Redescription of historic type material (Hartop et al. 2016) will likely prove to be an enormous help to those not working in close proximity to these definitive collections.

## Supplementary Material

XML Treatment for Megaselia baileyae

XML Treatment for Megaselia friedrichae

XML Treatment for Megaselia gonzalezorum

XML Treatment for Megaselia joanneae

XML Treatment for Megaselia losangelensis

XML Treatment for Megaselia phyllissunae

XML Treatment for Megaselia pongsaiae

XML Treatment for Megaselia shatesae

XML Treatment for Megaselia stoakesi

XML Treatment for Megaselia studentorum

XML Treatment for Megaselia voluntariorum

XML Treatment for Megaselia wongae

## Figures and Tables

**Table 1.** Species descriptions, *M. baileyae* - *M. phyllissunae*. Character remarks in parentheses, general remarks in last row.

	***Megaselia baileyae***	***Megaselia friedrichae***	***Megaselia gonzalezorum***	***Megaselia joanneae***	***Megaselia losangelensis***	***Megaselia phyllissunae***
**Head**						
SA ratio	0.46	0.77	0.65	0.49-0.59	0.80	0.47
VIF position	normal	normal	normal	normal	normal*	normal
SPS vesicles	absent	present	absent	absent	absent	absent
Palpal setae length	long	long	long	long	long	long
Labellum spinosity	spinose	spinose (sparse)	spinose	spinose (dense)	not	spinose
**Thorax**						
Anepisternum	bare	bare	bare	bare	bare	bare
Relative halter color	lighter	lighter	lighter	lighter	lighter (white)	lighter
# NP setae	2	2	2	2	2	2
NP cleft	absent	present (small)	absent	absent	present (short)	absent
Scutellar setae	2+2	2+2	2+2	2+2	2+2	2+2
**Leg**						
ts1 palisade	1-4	1-5 (5>4)	1-3 (fades out)	1-4	1-4	1-4
t2 palisade	0.74	0.6	0.8	0.75	0.80	0.75
t3 comb bifurcate	absent	absent	absent	absent	absent	absent
t3 setulae	PD	PD	PD	PD	PD	PD
f3 basal setae	f3>AV	f3>AV	f3>AV	f3<AV	f3>AV	f3>AV
f3 basal setae differentiation	absent	absent	absent	present(+/- 8 curved)	absent	present(+/- 8 curved)
**Wing**						
Wing Length (mm)	1.58	1.1	1.58-1.59	1.73	1.36	1.67
Subcosta	free	free	free	free	free (faint)	free
R seta	long (>3x vein)	long (~2x)	long (>3x vein)	short-long	long (4x+)	long (3x vein)
R2+3	present	present	present	present	present	present
Costal index	0.32-0.36	0.34-0.35	0.35-0.36	0.38-0.41	0.41	0.38
Costal ratios	4.7: 1.5: 1	4.0: 1.2: 1	4.95: 1.73: 1	3.5: 1.75: 1	3.68: 1.66: 1	3.5: 1.8: 1
Costal setae length (mm)	0.09	0.05	0.07	0.09	0.06	0.11
Number alular setae	3	2-3	2	2-4	2	2
Alular setae length (mm)	0.11	0.08	0.1	0.10-0.11	0.08	0.13
Wing color	lightly infuscated/ clear	lightly infuscated/ clear	lightly infuscated/ clear	lightly infuscated/ clear	lightly infuscated/ clear	lightly infuscated/ clear
**Genitalia**						
AT length	AT<E	AT+/-E	AT+/-E	AT+/-E	AT>=E	AT+/-E
E setation	hairs	hairs	hairs	hairs	hairs	hairs
General Remarks					*VFO high, upper SA wider than PO	

**Table 2.** Species descriptions, *M. pongsaiae* - *M. wongae*. Character remarks in parentheses, general remarks in last row.

	***Megaselia pongsaiae***	***Megaselia shatesae***	***Megaselia stoakesi***	***Megaselia studentorum***	***Megaselia voluntariorum***	***Megaselia wongae***
**Head**						
SA ratio	0.43	0.71	0.90	0.90	unequal*	0.76
VIF position	normal	normal	normal	normal	normal**	normal
SPS vesicles	absent	PP too dark to tell	absent	may be present	absent	may be present
Palpal setae length	long	long	long	long	long	long
Labellum spinosity	spinose (dense)	not	not	not	not	spinose (sparse)
**Thorax**						
Anepisternum	bare	bare	6-10 setae, 2 2x others	bare	bare	bare
Relative halter color	same	same	same	same	same	lighter
# NP setae	2	2	3	2	2	2
NP cleft	present (long)	absent	absent	absent	absent	absent
Scutellar setae	2+2	2+2	2+2	2+2	2+2	2+2
**Leg**						
ts1 palisade	1-4 (5?)	unknown	palisade cannot be determined, tarsi expanded with spinules	1-4	1-3 (fades out)	1-4
t2 palisade	0.67	0.67	0.63	0.62	0.57	0.67
t3 comb bifurcate	absent	absent	absent	absent	absent	absent
t3 setulae	PD	PD	PD	PD	PD	PD
f3 basal setae	f3>AV	f3>AV	f3<AV	f3>AV	f3+/-AV	f3>AV
f3 basal setae differentiation	absent	present (+/- 9, curved)	present*	absent	absent	absent
**Wing**						
Wing Length (mm)	1.5	1.9	1.65	1.55	1.09	1
Subcosta	free	complete	free	complete	complete	free
R seta	long (4x+)	small	absent	long (2x+)	short	long (slightly > 2x vein)
R2+3	present	present	present	present	present	present
Costal index	0.40	0.38	0.40-0.41	0.52	0.38	0.37-0.40
Costal ratios	4.5: 1.86: 1	3.7: 1: 1	4.2: 2.25: 1	2.4: 1.85: 1	3.04: 1.6: 1	3.4: 1.15: 1
Costal setae length (mm)	0.08	0.18	0.10	0.14	0.08	0.06
Number alular setae	2	5	3	4	2-3	2
Alular setae length (mm)	0.1	0.13+ (angled on slide)	0.10-0.11	0.12	0.08	0.08
Wing color	lightly infuscated/ clear	lightly infuscated/ clear	lightly infuscated/ clear	lightly infuscated/ clear	lightly infuscated/ clear	lightly infuscated/ clear
**Genitalia**						
AT length	AT<E	AT<E	AT<E	AT<E	AT<E	AT<E
E setation	hairs	hairs	bristles (weak)	hairs	hairs	hairs
General Remarks			*F3 with short, blunt spines basally	large C2	*upper SA missing **close to VFO	
